# Vicarious pain is an outcome of atypical body ownership: Evidence from the rubber hand illusion and enfacement illusion

**DOI:** 10.1177/17470218211024822

**Published:** 2021-06-29

**Authors:** Vanessa Botan, Abigail Salisbury, Hugo D Critchley, Jamie Ward

**Affiliations:** 1 School of Psychology, University of Sussex, Brighton, UK; 2 Sackler Centre for Consciousness Science, Brighton, UK; 3 Brighton and Sussex Medical School, Brighton, UK

**Keywords:** Vicarious pain, rubber hand illusion, embodiment, time perception, enfacement illusion

## Abstract

Some people report localised pain on their body when seeing other people in pain (sensory-localised vicarious pain responders). In this study, we assess whether this is related to atypical computations of body ownership which, in paradigms such as the rubber hand illusion (RHI), can be conceptualised as a Bayesian inference as to whether multiple sources of sensory information (visual, somatosensory) belong together on a single body (one’s own) or are distributed across several bodies (vision = other, somatosensory = self). According to this model, computations of body ownership depend on the degree (and precision) of sensory evidence, rather than synchrony per se. Sensory-localised vicarious pain responders exhibit the RHI following synchronous stroking and—unusually—also after asynchronous stroking. Importantly, this occurs only in asynchronous conditions in which the stroking is predictable (alternating) rather than unpredictable (random). There was no evidence that their bottom-up proprioceptive signals are less precise, suggesting individual differences in the top-down weighting of sensory evidence. Finally, the enfacement illusion (EI) was also employed as a conceptually related bodily illusion paradigm that involves a completely different response judgement (based on vision rather than proprioception). Sensory-localised responders show a comparable pattern on this task after synchronous and asynchronous stroking. This is consistent with the idea that they have top-down (prior) differences in the way body ownership is inferred that transcends the exact judgement being made (visual or proprioceptive).

## Introduction

The sense of self primarily arises from the feeling of one’s body (Costantini, 2014; Tsakiris, 2017). Notably, the experience of our bodily self is not always definite or coherent as revealed by bodily illusions on neurotypical participants (e.g., Blanke & Metzinger, 2009; Botvinick & Cohen, 1998; Tsakiris, 2008) or neurological symptoms following brain damage such as somatoparaphrenia (denial of limb ownership; Feinberg et al., 2009). Even within the general (neurotypical) population, there are likely to be substantial differences in the phenomenological experience of the bodily self that are underpinned by individual differences in the fidelity of relevant bodily signals and/or differences in the way that these signals are evaluated. One relevant group are people who report feeling the pain of others (termed vicarious pain responders; Grice-Jackson et al., 2017; Osborn & Derbyshire, 2010). For these people, observations of pain elicit a pain-like phenomenology on their own body (and often a mirroring of other kinds of sensations and feelings; Fitzgibbon et al., 2010). Thus, observed bodily experiences on other people are jointly shared between self and other, and this can arguably reflect a misattribution of body ownership (Ward & Banissy, 2015). In this view, vicarious pain can act as a marker of important neurocognitive differences in body perception (beyond the defining symptom of vicarious pain itself). The present study uses presence/absence of vicarious pain to address key questions relating to the mechanisms of body ownership and its variability within the general population. Two paradigms relating to body ownership and self–other judgements are used: the rubber hand illusion (RHI) and the enfacement illusion (EI).

The most popular paradigm proving the malleability of bodily ownership is the RHI (Botvinick & Cohen, 1998). In this paradigm, participants tend to report ownership over a dummy hand thus expanding their own bodily boundaries. The paradigm consists in placing a dummy hand in front of the participants while their real hand is hidden from view. Subsequently, both hands are stroked either synchronously (at the same time) or asynchronously (typically out of phase), and most evidence shows that the illusion is stronger in the synchronous condition (Botvinick & Cohen, 1998; Tsakiris & Haggard, 2005). The illusion is reflected in an objective measure termed proprioceptive drift: participants report that the real hand is positioned closer to the rubber hand. It is also reflected in subjective measure using questionnaires: participants report experiences of ownership, self-location, or agency over the fake hand.

The main theoretical explanation states that body ownership is inferred when external sensory inputs match each other (e.g., synchronous stroking) and also when they match the internal representation of the body, such as the orientation of the hand (Costantini & Haggard, 2007). An alternative explanation is that the RHI is disrupted by asynchrony rather than elicited by synchrony (Rohde et al., 2011). Given that both accounts make the same prediction with regard to the standard RHI conditions (i.e., synchronous > asynchronous), additional conditions are needed to adjudicate between them. For instance, merely observing a dummy hand with no stroking to either the dummy or own hand (i.e., no temporal or tactile cues, only visuo-spatial cues) could act as a baseline. This vision-only condition also elicits the RHI, at least in terms of proprioceptive drift, albeit not as strong as the synchronous condition (Rohde et al., 2011; Samad et al., 2015). This suggests that while the RHI is influenced by temporal signals (both enhanced by synchronous cues and disrupted by asynchronous ones), it is not critically dependent on them (see also Durgin et al., 2007). Samad et al. (2015) applied the Bayesian causal sensory inference model to explain these findings. This framework argues that the RHI derives from the perception of a common cause for sensory signals (proprioceptive, tactile, and visual). This can also be construed as a judgement as to whether the sensory signals belong to the same body (one’s own) or not (hence, body ownership is a particular kind of causal inference). Incoming sensory evidence is contrasted with prior probabilities (e.g., the probability that the touch that I feel is caused by the touch that I see), to estimate the likelihood of a common cause. Synchrony and asynchrony provide evidence for and against a common cause. In the absence of temporal cues, the mere visual presence of an appropriate hand in peripersonal space constitutes supportive evidence of common cause. This occurs because, in this model, visual information is weighted more strongly than proprioceptive information owing to the fact that vision is expected to be precise (termed *higher precision weighting*) even though vision is misleading in this context. In Bayesian-related predictive processing models, the discrepancy between the sensory evidence and the model (i.e., the belief in common cause or not) is termed a prediction error and the system seeks to minimise this. Proprioception always provides evidence against a common cause (because the felt position of the limb contradicts the observed limb position) and would generate a prediction error. To resolve the prediction error, there are two options: either to reject the model of common cause (i.e., believe the proprioceptive signal) or to shift the sensory evidence to make it fit better with a common cause (i.e., generate a proprioceptive drift).

In this Bayesian inference framework, there are multiple ways in which individual differences (e.g., due to normal variation or clinical or neurological conditions) can manifest themselves. For instance, some people may differ in the reliability of sensory evidence from one or more channels and, specifically, this framework predicts that people will “play to their strengths” (i.e., weight precise evidence more strongly; Ernst & Banks, 2002). For instance, someone with good proprioceptive abilities (who knows precisely where their limbs are in space) should weight this information strongly and be less susceptible to the illusion (and vice versa for someone with poor proprioception). Other people may have biases within their internal model, such as a greater tendency to perceive common causes given ambiguous evidence (a trait linked to Schizophrenia; for example, Tschacher & Kupper, 2006) or greater top-down expectancies of percept-like experiences (“phenomenological control”; Lush et al., 2020). In a recent study, Botan et al. (2018b) reported an atypical pattern in the RHI in a group of people who report sensory-localised (S/L) vicarious pain (i.e., they feel pain when seeing others in pain localised to the same body part). This pattern consisted of experiencing the RHI both in the synchronous and in the asynchronous conditions. This pattern has very rarely been reported in the literature (but see Kaplan et al., 2014; Zopf et al., 2016) and runs against one of the central dogmas in this field (i.e., synchronous > asynchronous). One might wonder whether this reflects a general tendency to report the RHI under all conditions, but Botan et al. (2018b) showed that this was not the case. They reported two control conditions that did not elicit an RHI in any group: one in which the dummy is stroked but not the real hand, and the converse condition of stroking the real hand but not the dummy. In this study, we seek to replicate and extend these observations. Specifically, we note that in the most commonly used asynchronous condition of the RHI, the visual and tactile signals are correlated and predictable: the strokes are alternated at a fixed temporal lag. In other paradigms, correlated sensory signals with a fixed temporal lag are integrated together (at least at short lags), which is assumed to reflect an inference of common cause (Fujisaki et al., 2004; Keetels & Vroomen, 2008; Parise et al., 2012). Hence, it is plausible that—in some people at least—this kind of correlated asynchronous signal can elicit the RHI. To test this hypothesis, we introduce a second kind of asynchronous stroking (termed asynchronous-random) in which the alternate strokes vary in speed, length, and duration, so there is no fixed or predictable lag. We also introduce a vision-only condition in which no stroking is applied to either hand, with the aim to replicate previous findings that this condition can elicit an RHI (Rohde et al., 2011; Samad et al., 2015) and to determine whether this effect is more pronounced in our vicarious pain group. The latter would suggest a mechanism of visual capture, that is, an unusually strong discounting of proprioceptive signals in favour of visual evidence. Finally, we measure individual differences in the variability of proprioceptive signals at baseline to determine whether people with more variable proprioceptive signals (less precision) are more susceptible to the RHI and whether this can account for any group differences.

A second paradigm that has been widely used to investigate the bodily self is the EI, a facial analogue of the RHI, which uses tactile stimulation of the face to manipulate the perceived similarity with another unfamiliar person (Sforza et al., 2010; Tsakiris, 2008). The participants’ face is stroked while they see somebody else stroked on their face either synchronously or asynchronously, and the dependent measure is a judgement of visual appearance (not proprioception). Namely, participants are presented with morphed faces—morphed between their own face and the face of the stroked person—and they have to judge the transition point at which the morph begins to resemble the other person’s face more than their own (termed point of subjective equality [PSE]). After synchronous stroking, the PSE is shifted from self towards other. In effect, it is as if participants have updated their own internal model of self-appearance to fit the sensory evidence, that is, a recalibration of visual appearance to fit a common cause inference that the person that they saw being stroked was themselves. Subjectively, they also report feeling greater ownership and agency over the model’s face (Tajadura-Jimenez et al., 2011). In this study, the EI complements the RHI in two important aspects. First, it explores malleability over a face, and not only a hand. Second, it serves to clarify the role of proprioception in the susceptibility to bodily ownership paradigms. If vicarious pain responders have particular difficulties in knowing where their body is in space or tend to weight that kind of information in an idiosyncratic way, then we would expect all groups to behave similarly (i.e., have the same PSEs and the same effect of synchrony > asynchrony). However, if there are individual differences in higher order judgements of bodily self–other (or body-based causal inferences more generally), then we expect a similar pattern between both paradigms.

Having outlined the overarching rationale behind the research, it would be useful to provide more detailed background on the special population under investigation, namely, vicarious pain responders. This is conceptually related to mirror-touch synaesthesia (feeling touch on one’s own body when seeing other people touched), although vicarious pain, or “mirror pain,” experiences tend to be far more common being found in around ~20% of the population (Grice-Jackson et al., 2017; Osborn & Derbyshire, 2010). Others have used the overarching term *mirror-sensory synaesthesia* to describe both of these phenomena (Fitzgibbon et al., 2010). These are associated with various functional (functional magnetic resonance imaging [fMRI]) and structural (voxel-based morphometry [VBM]) brain differences that corroborate the fact that these individuals are different (Grice-Jackson et al., 2017; Holle et al., 2013). These include, but are not limited to, regions involved in somatosensation. For instance, there is evidence of reduced grey matter in the right temporoparietal junction (Grice-Jackson et al., 2017; Holle et al., 2013), a region that is implicated in selectively attending to self versus other (Bird & Viding, 2014) and that has been implicated in the RHI (Tsakiris, 2010). Grice-Jackson et al. (2017) developed a measure termed the Vicarious Pain Questionnaire (VPQ), which presents participants with a series of videos (of injections and sporting accidents) and requires participants to report, quantify (intensity), and describe (using pain and location descriptors) any pain experiences in their own body. Using clustering techniques, three groups were identified. The most common group reported few if any pain-like experiences (non-responders or controls), and two groups frequently reported pain experiences: S/L responders reported localised pain experiences using sensory descriptors, whereas A/G (affective-general) responders reported non-localised, or whole body, experiences using mainly affective descriptors. The S/L group is more closely linked to mirror-touch (Ward et al., 2018), and only this group was reported to have the atypical pattern (on asynchronous stroking) in the RHI (Botan et al., 2018b). Hence, our main hypotheses only relate to this S/L group relative to non-responder controls. A smaller A/G group is tested for completeness and they are predicted to be normal on this measure (i.e., similar to non-responders). (A meta-analytic sample—combining data from this study with Botan et al., 2018b—is included in the Supplementary Results for the synchronous and asynchronous conditions that are common to both studies.)

## Method

### Participants

A total of 59 participants (*M* age = 22.28, *SD* = 4.53; 49 females) took part in the study. Participants were recruited from the student population at Sussex University, and all but four (2 S/L and 2 A/G) had never taken part in our previous research on the RHI (Botan et al., 2018b). Ethical approval was obtained from the Science and Technology Research Ethics Committee of the University of Sussex, and all participants offered their written informed consent at the beginning of the study.

There were 27 participants classed as non-responders (i.e., controls; *M* age = 23.26, *SD* = 5.64; 19 females), 20 participants classed as S/L responders (*M* age = 21.52, *SD* = 3.62; 17 females), and 12 participants classed as A/G responders (*M* age = 21.42, *SD* = 2.64; 11 females). Although the latter group is smaller, it is to be noted that this group was not crucial to our hypotheses (which focussed on the S/L group). The groups did not differ by age, *F*(2, 57) = 1.144, *p* = .326, η^2^ = 0.039, or gender (χ² = 2.351, *p* = .309). In the RHI task, eight participants (six controls and two S/L) lack measurements for proprioceptive variance and the asynchronous-random condition, which were introduced at a later time. They were still included in the analysis of the other conditions. Due to technical and logistical issues, seven participants (four controls and three S/L) did not complete the EI task.

### VPQ

Before completing the tasks, all participants undertook the VPQ. They watched 16 videos (no audio) of people experiencing physical pain (e.g., falls, sports injuries, injections; Grice-Jackson et al., 2017). After each video, they had to report (1) whether they experienced a bodily sensation of pain, (2) how intense was that pain (1–10 Likert-type scale), and (3) whether the pain was localised to the same place, to a different place, or generalised to the entire body, and they were asked to describe the pain selecting various pain adjectives. These answers were used to generate the three variables (i.e., pain intensity, localised-generalised responses, and sensory–affective responses) and entered the two-step cluster analysis conducted on a larger dataset of participants (aged 18–60 years, *M* age = 20.11, *SD* = 6.94; 290 males, 1,004 females). For further details, see Botan et al. (2018b).

### RHI

This study used an identical procedure to Botan et al. (2018b) for synchronous and asynchronous conditions but additionally considered two new conditions (visual-only and asynchronous-random).

#### Materials

In the RHI task, participant’s right arm was placed in a box (86 cm across × 60 cm wide × 20 cm high) with a dummy right hand placed 20 cm to the left of it (measured from the two index fingers) at the body midline. The top surface of the box had a section cut out of it so that only the dummy hand was visible during the experimental manipulations. The top surface was entirely covered for the proprioceptive judgements and illusion ratings.

#### Procedure

At the beginning of each condition, the participant was asked to estimate the location of her or his right index fingertip three times by reading the corresponding number along a 1-m ruler; the offset of the ruler varied each time to prevent repetition of the same number. This generated 12 baseline location measurements (three for each of the four conditions). The standard deviation was calculated for each participant across the 12 measurements, giving a measure of proprioceptive imprecision. That is, a higher score indicates greater instability in knowing the location of one’s own hand.

Four conditions were performed in a counterbalanced order across participants, each lasting for 2 min: synchronous (the timing of the brush strokes on the rubber hand and participant’s own hand was synchronised), asynchronous (the timing of the brush strokes was out of phase by approximately 625 ms), vision-only (no stroking at all, the participants had to look at the rubber hand for 2 min), and asynchronous-random (the timing of the brush strokes was out of phase, but this time was completely random; the participants could not predict when the next stroke would start or whether it would be fast or slow). The stroking was always applied to the index finger using paintbrushes.

Post-induction finger location judgements were obtained in the same manner as the initial baseline. Proprioceptive drift was calculated by subtracting the average of the pre-induction finger location judgements from the average of the post-induction finger location judgements.

After each condition, participants completed the RHI questionnaire comprising 10 items divided into three subscales: Ownership, Location, and Agency (Longo et al., 2008; see Supplementary Materials and Results S1.1 for further details). The items were measured on a 7-point Likert-type scale (1 = *strongly disagree*, 7 = *strongly agree*).

### EI

This was closely based on previously published procedures (Maister et al., 2013; Tajadura-Jimenez et al., 2011).

#### Materials

The EI task comprised 120-s long clips showing the face of a model being stroked on the right cheek with a cotton bud at a frequency of approximately one stroke per second. There were four models: two females and two males. Digital photographs of their faces were taken and subsequently edited in Photoshop CS6, removing all non-facial attributes (i.e., hair, ears, etc.) using an oval mask and superimposing it on to a uniform grey background. Both clips and photographs were in black and white, and the models had a neutral expression. The models and participants were gendered-matched, and both models were Caucasian (N.B. race does not seem to influence the illusion; Bufalari et al., 2014).

Prior to the experiment, a photograph of the participant face was also taken and edited following the same procedure as the models’ photographs. Subsequently, the participant face was morphed into the model face using the Abrasoft FantaMorph 5 software. The procedure generated morphs of 2% increments in which the participant’s face was merged with the model face resulting in 50 pictures per model (Sforza et al., 2010). The morphs used in the task varied between 30% and 70% resulting in a total of 21 morphed pictures, the first picture representing 30:70 model:participant and the last picture 70:30 model:participant.

#### Procedure

The experiment consisted of two blocks—synchronous and asynchronous—the order of which was counterbalanced across participants. A different model was used in the asynchronous and synchronous blocks to minimise any spillover of behavioural effects across blocks, and assignment was counterbalanced across participants (but the model was always the same sex as the participant).

**Figure 1.**
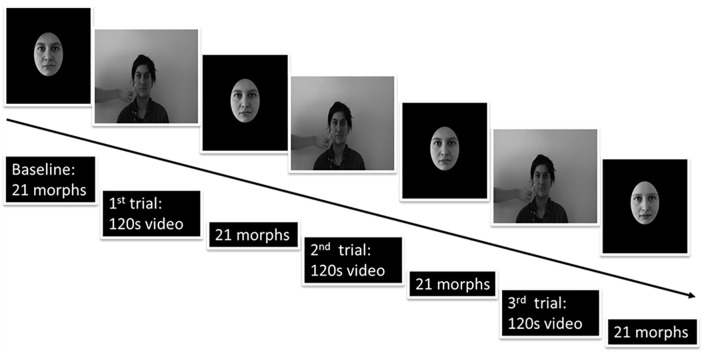
EI task detailed representation.

A detailed representation of the task can be seen in Figure 1. At the start of each block, participants performed a baseline self-recognition task. All 21 morphs were shown in a randomised order, and participants had to judge if the face looked “more like myself” or “more like the model’s face” (using left and right arrow keypresses, respectively). The movie depicting stroking to the model’s face was then shown while the participants’ own face was stroked, in the mirrored location, either synchronously (in phase) or asynchronously (out of phase by ~500 ms). This was followed by another self-recognition task, with the movie-task cycle repeated three times in total.

At the end of each block, participants completed the EI questionnaire (Tajadura-Jimenez et al., 2011). This comprised 14 items divided into four subscales: Ownership, Appearance, Disownership, and Agency (see Supplementary Materials and Results S1.2 for further details). The items were measured on a 7-point Likert-type scale (1 = *strongly disagree*, 7 = *strongly agree*).

#### Analysis

For each self-recognition task, the PSE, representing the point when the participants cannot distinguish between self and other, was calculated using a logistic function (Bacaër, 2011). The logistic function was applied to the percentages of the morph data (*x* values) generating binary probabilities of *y* fitted values (Cramer, 2003). The *x* value corresponding to the minimum value of the sum of square differences between the *y* values (actual binary responses) and *y* fitted values (the binary probabilities generated by the logistic function) represented the PSE, namely, the steep transition of the sigmoid curve. For each condition, a PSE drift score was calculated by averaging the three experimental PSEs and then subtracting from the appropriate initial baseline. For example, a value of +6 would mean that the experimental manipulation had shifted the PSE away from the self towards the other by 6% of the morphed images, whereas a negative PSE drift value would imply that the person resembles the other person *less* as a result of the experimental manipulation.

### Data analysis

All analyses were conducted in SPSS, Version 25 (SPSS, Inc., Armonk, NY, USA). Synchronous and asynchronous conditions for objective measures of proprioceptive drift in the RHI and PSE in the EI were analysed using 3 (group) × 2 (condition) mixed-model analyses of variance (ANOVAs).

The other dependent measures in the RHI task including proprioceptive imprecision, vision-only condition, and asynchronous-random condition were analysed using between-group one-way ANOVAs. Most variables passed the Shapiro–Wilk normality tests and homogeneity of variance tests, the only exceptions being the vision and asynchronous-random drift in the S/L group (*p* < .05). The Pearson correlations between proprioceptive imprecision and drift magnitude in each condition were also run.

Questionnaire results for both RHI and EI were analysed using non-parametric Kruskal–Wallis *H* tests for ordinal data comparing all groups and subsequent post hoc non-parametric Mann–Whitney *U* tests comparing two independent groups.

Outliers were excluded for each condition using SPSS based on the third interquartile range (3-IQR; Manikandan, 2011). Thus, one outlier was excluded from the asynchronous condition, four from the vision-only condition, one from the asynchronous-random condition, and three from proprioceptive imprecision. No outliers were found in the questionnaire data outside the 3-IQR. Subsequent post hoc tests adjusted for multiple comparisons (Bonferroni corrections) assessed differences between and within groups.

## Results

This section first addresses the results of the RHI first by considering how proprioceptive drift differs by condition across the groups, whether this is related to individual differences in proprioceptive imprecision, and finally self-reported illusory experiences. The results of the EI task report the PSE and questionnaire measures in that order.

### RHI: proprioceptive drift

**Figure 2.**
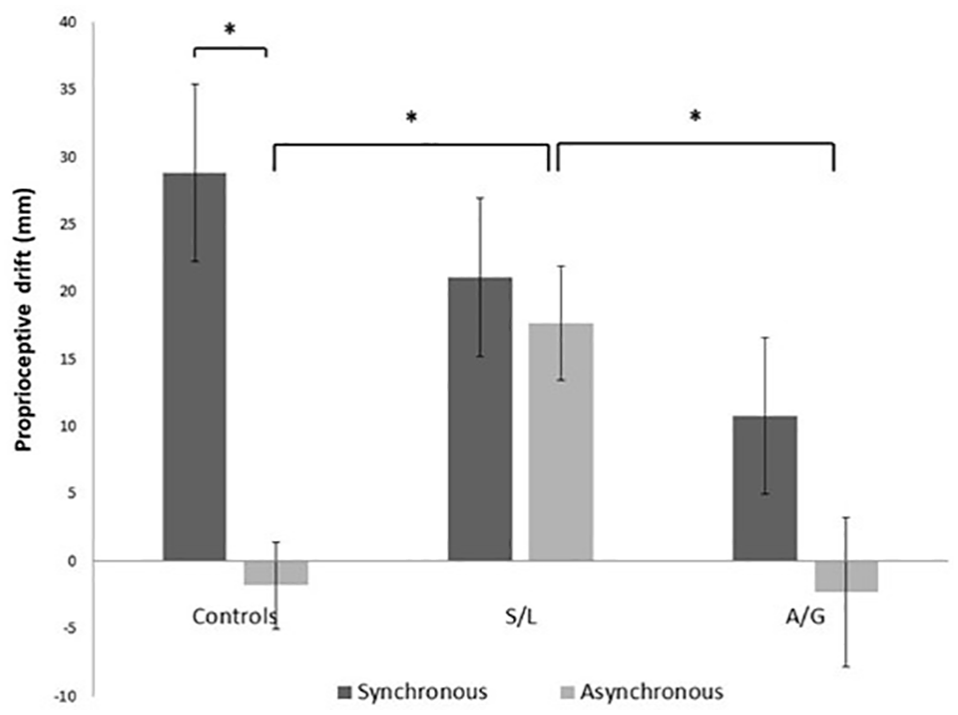
Proprioceptive drift in the synchronous and asynchronous conditions in each group. S/L: sensory-localised; A/G: affective-general. Bars indicate *M* ± 1 *SE*. *p < .05.

The results for the standard RHI synchronous and asynchronous conditions are shown in Figure 2. A mixed-model 3 (group) × 2 (condition) ANOVA run on the synchronous and asynchronous conditions showed a statistically significant Group × Condition interaction, *F*(2, 54) = 3.756, *p* = .030, η^2^ = 0.122. There was a main effect of condition, *F*(1, 54) = 7.918, *p* = .007, η^2^ = 0.128, but the main effect of group did not reach significance, *F*(2, 54) = 2.006, *p* = .144, η^2^ = 0.069. Post hoc paired *t* tests revealed that proprioceptive drift was significantly greater in the synchronous condition than in the asynchronous condition in the control group, *t*(26) = 4.268, *p* < .001, but not in the S/L group, *t*(18) = 0.453, *p* = .656, nor in the A/G group, *t*(10) = 0.888, *p* = .395. For the asynchronous condition, independent *t* tests revealed a significant higher drift in the S/L group when compared with the control group, *t*(44) = –3.621, *p* = .001, and the A/G group, *t*(29) = 2.474, *p* = .019. This replicates the findings of Botan et al. (2018b) for these groups and these conditions.

**Figure 3.**
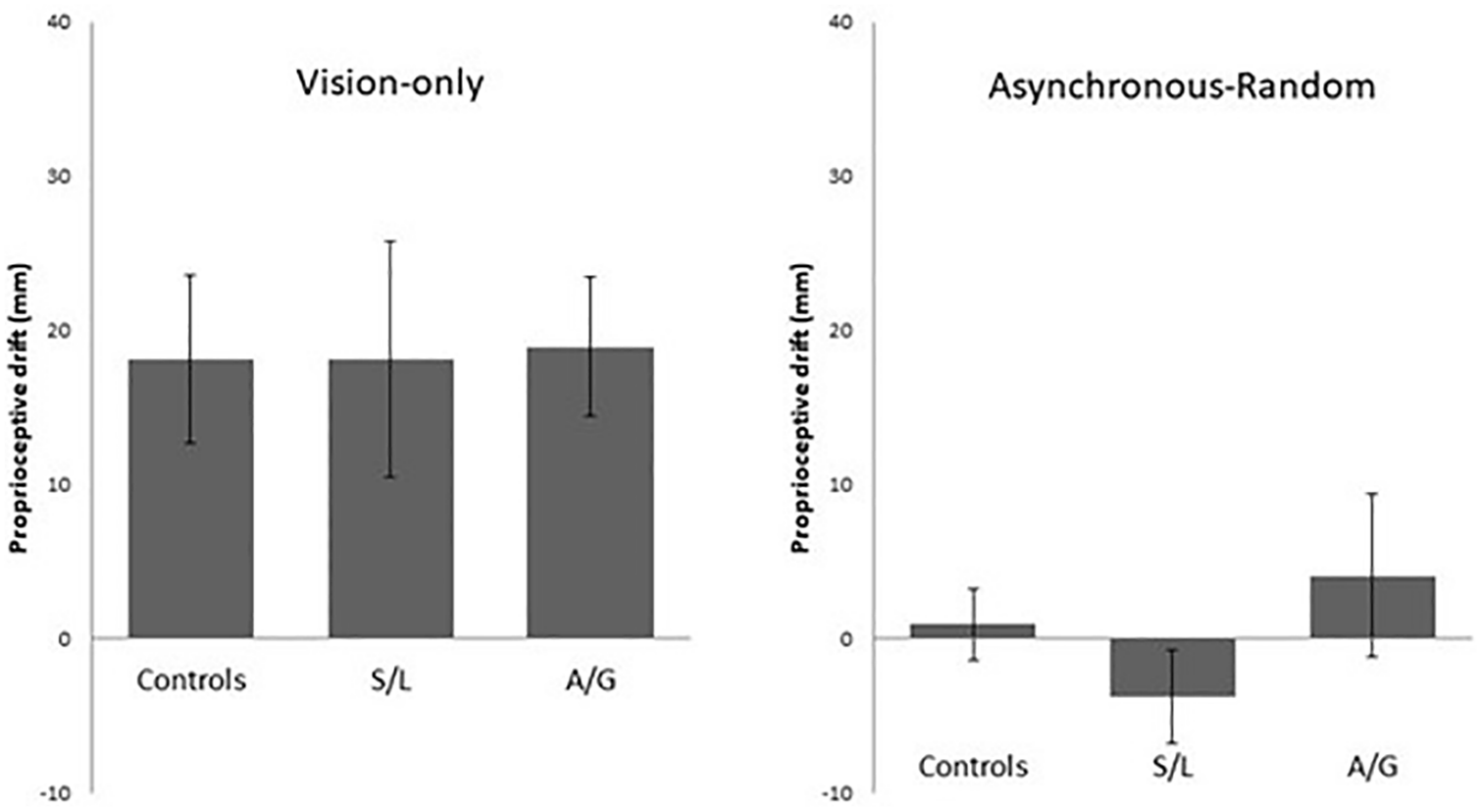
Proprioceptive drift in the vision-only condition (left) and asynchronous-random condition (right). S/L: sensory-localised; A/G: affective-general. Bars indicate *M* ± 1 *SE*.

The results for the two novel conditions are shown in Figure 3. No significant group differences were found in the vision-only condition, *F*(2, 56) = 0.008, *p* = .992, η^2^ = 0.015, or in the asynchronous-random condition, *F*(2, 44) = 1.239, *p* = .300, η^2^ = 0.003. To test whether the illusion is induced at all in these conditions, relative to an a priori baseline drift of zero, one-sample *t* tests were conducted. The vision-only condition also resulted in the RHI for all groups (one-sample *t*s of 3.342, 2.266, and 4.003; all *p*s < .05 for controls, S/L, and A/G), as reported by Rohde et al. (2011). By contrast, the asynchronous-random condition produced no RHI in any group (one-sample *t*s of 0.962, –1.090, and 0.739; all *p*s > .1 for controls, S/L, and A/G). Thus, the tendency for the S/L group to experience the RHI in the standard asynchronous condition does not appear due to increased visual capture, but instead appears to depend on the predictable temporal lag between vision and touch.

**Figure 4.**
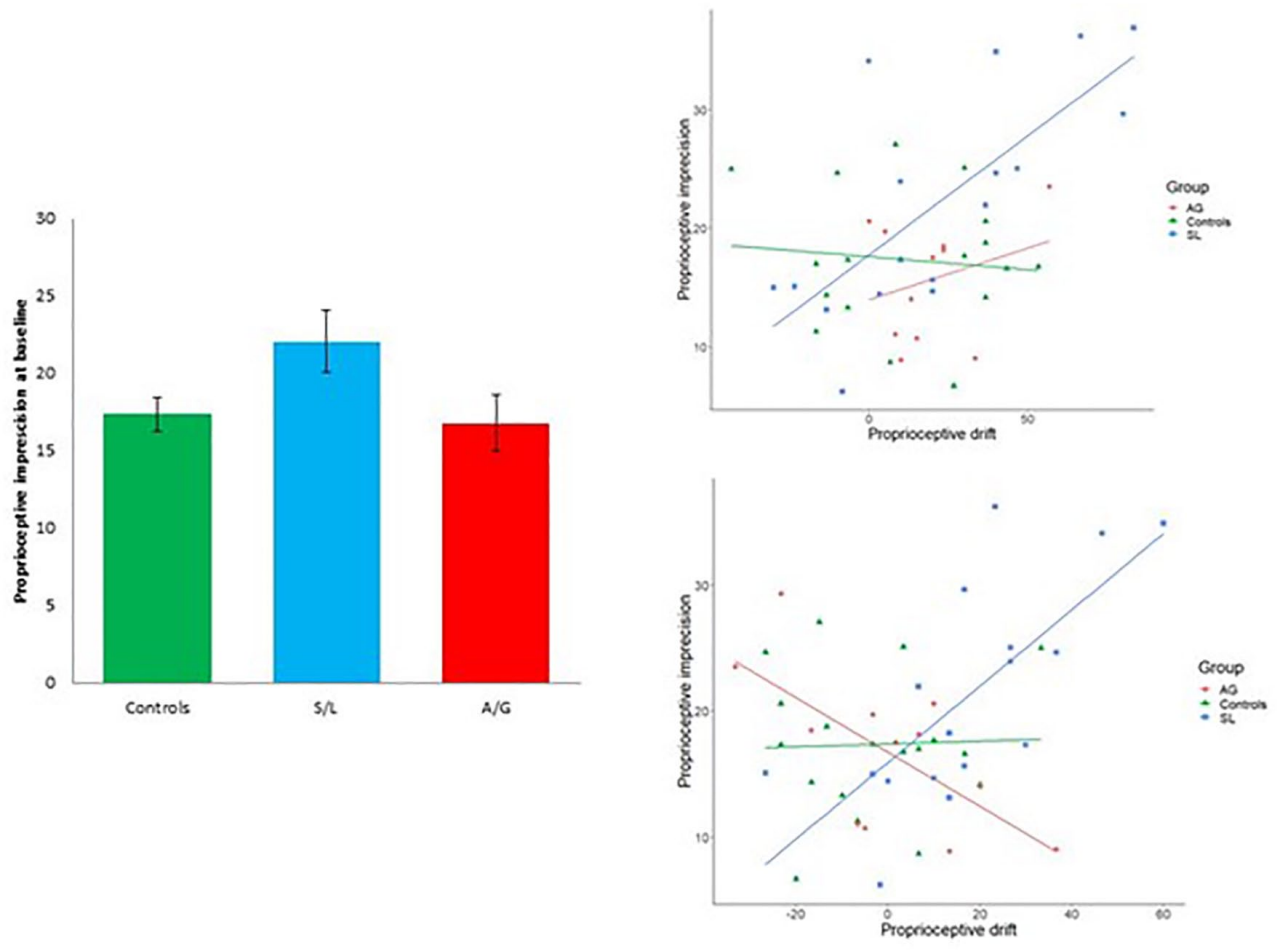
Left: Mean proprioceptive imprecision at baseline expressed in millimetres (bars show 1 SEM); Top right: Correlation between proprioceptive imprecision at baseline and drift in vision-only condition; Bottom right: Correlation between proprioceptive imprecision at baseline and drift in the asynchronous condition.

The results for the measure of proprioceptive imprecision (i.e., standard deviation of proprioceptive judgements at baseline) are shown in Figure 4. There were no significant differences between groups, *F*(2, 49) = 2.705, *p* = .077, η^2^ = 0.086. In terms of individual differences across the entire sample, proprioceptive imprecision at baseline was linked to greater proprioceptive drift at test in two conditions: the asynchronous-predictable, *r* = .301, *p* = .036, and vision-only, *r* = .444, *p* = .002, conditions (the correlations for synchronous and asynchronous-random conditions are .087 and .097, respectively). That is, in the two most ambiguous conditions, participants’ proprioceptive abilities have a stronger influence: people with less precise proprioception tend to discount proprioceptive information and generate a stronger RHI. Breaking this down by group shows that the effect is driven by the S/L group (for the asynchronous condition, *r* = .716, *p* = .001; for the vision-only condition, *r* = .731, *p* = .001). The full set of correlations by group is reported in the Supplementary Results. Thus, while the S/L group does not have higher proprioceptive imprecision, it is more susceptible to its influence. The data presented below, from the EI, where proprioception is irrelevant, suggest that differences in proprioception cannot account for the overall effect on the asynchronous condition.

### RHI: subjective ratings

**Figure 5.**
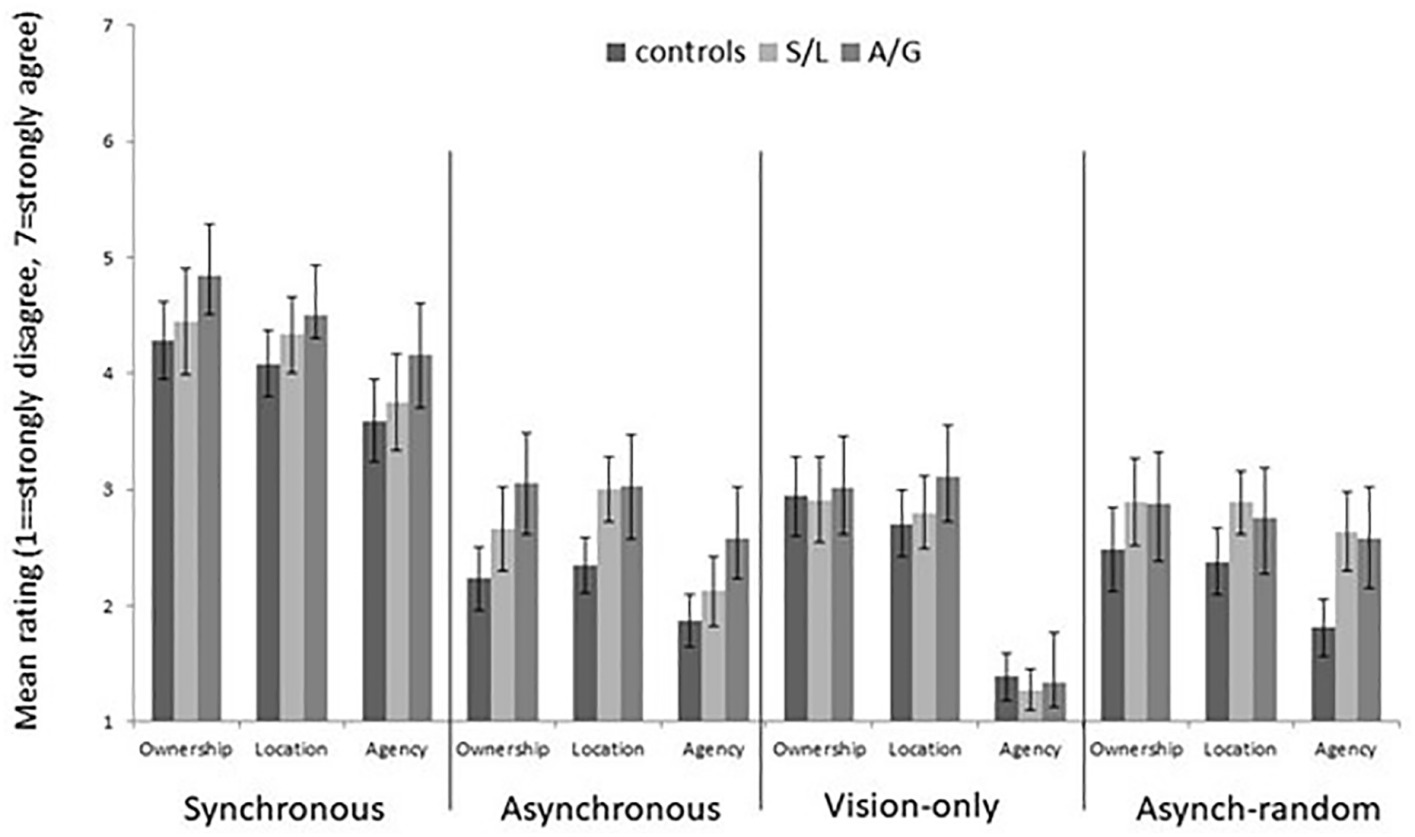
Mean subjective ratings for the RHI questionnaire according to group and subscale. Error bars show 1 SEM.

The questionnaire data for the four conditions, and different scales, are shown in Figure 5. Non-parametric Kruskal–Wallis *H* tests for ordinal data were used to analyse differences between groups for each condition and on each of the subscales. There were no significant differences between groups on any of the conditions or subscales (see Supplementary Results for the full breakdown). In particular, it is to be noted that there were no significant group differences on the asynchronous condition that mirror those reported above for proprioceptive drift. Previous findings show that these two measures can sometimes be dissociated suggesting that different mechanisms contribute to drift versus illusory experience (e.g., Holle et al., 2011; Holmes et al., 2006). Within our dataset, there is other evidence consistent with this. Across all groups, there is a significant proprioceptive drift in the vision-only condition, but this is not reflected in the subjective ratings which resemble both asynchronous-random and asynchronous-predictable conditions in this regard (a mean score <4 on this 1–7 scale indicates net disagreement with these statements).

### EI: PSE

**Figure 6.**
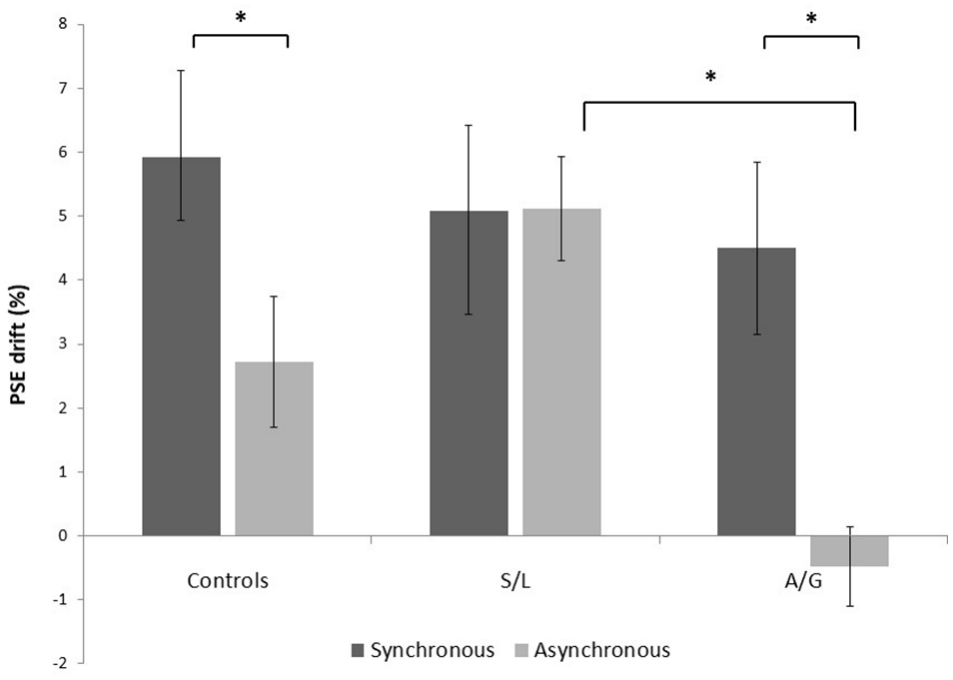
PSE drift relative to baseline for synchronous and asynchronous conditions in each group. S/L: sensory-localised; A/G: affective-general. Bars indicate *M* ± 1 *SE*. *p < .05.

These results are summarised in Figure 6. PSE drifts for the synchronous and asynchronous blocks were analysed in a mixed-model 3 (group) × 2 (condition) ANOVA. The results showed a statistically significant interaction between group and condition, *F*(2, 50) = 3.418, *p* = .041, η^2^ = 0.120. There was a main effect of condition, *F*(1, 50) = 12.363, *p* = .001, η^2^ = 0.198, and no significant main effect of group *F*(2, 50) = 2.271, *p* = .114, η^2^ = 0.083. Post hoc paired *t* tests revealed that PSE drift, relative to baseline, was significantly greater in the synchronous condition than in the asynchronous condition in the control group, *t*(22) = 3.850, *p* = .001, and in the A/G group, *t*(11) = 3.561, *p* = .004, but not in the S/L group, *t*(17) = –0.022, *p* = .983. Independent *t* tests revealed a significant higher drift for the asynchronous condition in the S/L group when compared with the A/G group, *t*(28) = –5.024, *p* = .001, but not the control group *t*(39) = –1.749, *p* = .088.

### EI: subjective ratings

**Figure 7.**
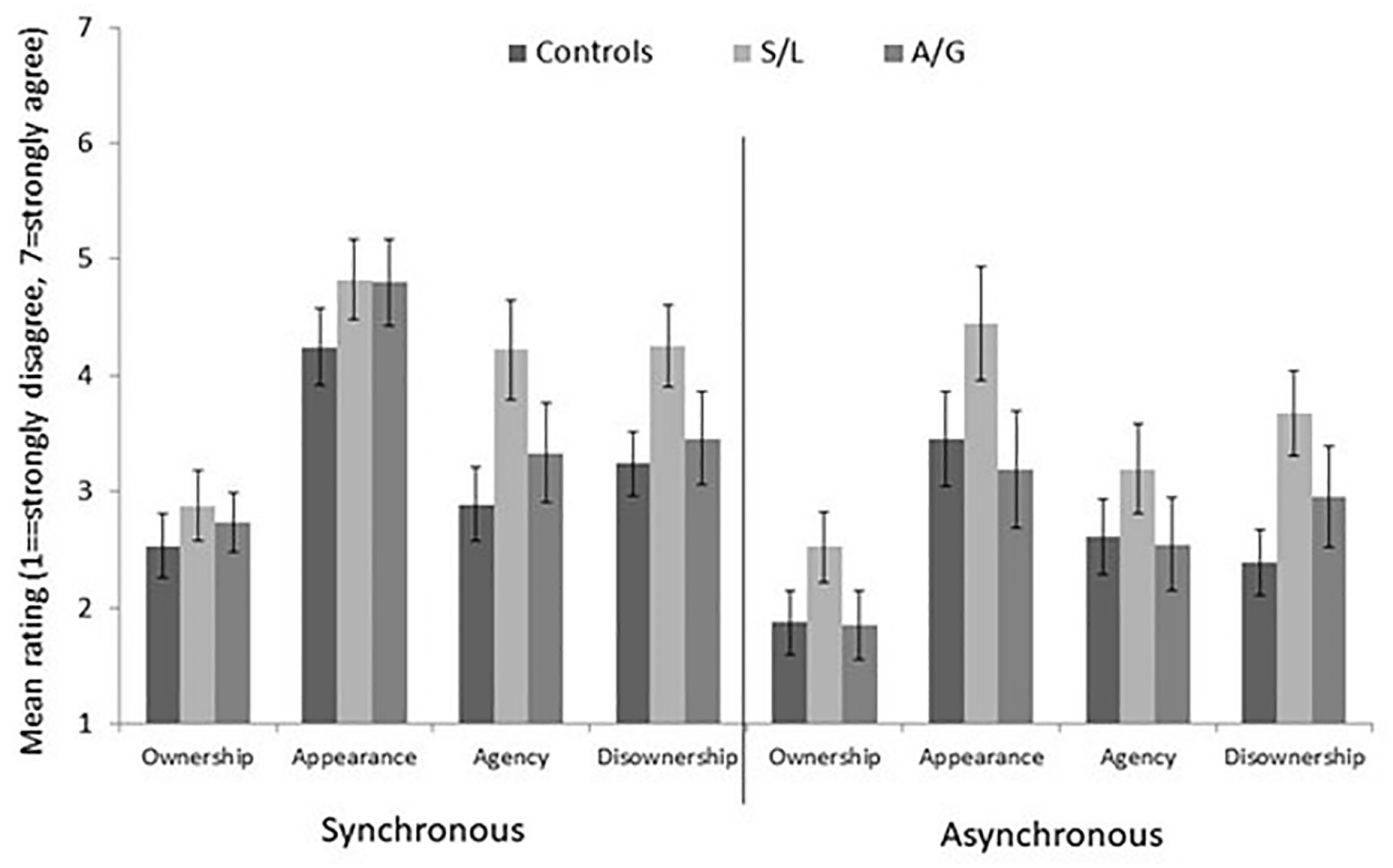
Mean subjective ratings for the EI questionnaire according to group and subscale. Error bars show 1 SEM.

The results are summarised in Figure 7. Non-parametric Kruskal–Wallis *H* tests for ordinal data were used to analyse differences between groups for each condition and on each of the subscales. There were no significant differences between groups on most of the conditions or subscales except for the Disownership subscale in the asynchronous condition. Mann–Whitney *U* test revealed that the S/L group reported greater disownership in the asynchronous condition than the control group, *Z* = –2.634, *p* = .008. For instance, they were more likely to endorse statements such as “The experience of my own face was less vivid than normal.”

## General discussion

The aim of this study was to use vicarious pain responders as a model system to explore individual differences in the computation of body ownership, in two well-established illusions: the RHI and the EI. These tasks can be construed, within a Bayesian Sensory Inference framework (Samad et al., 2015), as an inference as to whether different sensory signals reflect a common cause (with visual and bodily signals reflecting a single event bound to their own body) or different causes (multiple events across self and other). Mirror-sensory synaesthesia, which includes mirror-touch and S/L vicarious pain, has been hypothesised to reflect a disruption of self–other control resulting in self-attribution of other people’s experiences (Ward & Banissy, 2015). The present study sheds new light on the specific mechanisms that might underpin this. Specifically, there was a greater tendency to infer body ownership (common cause) after asynchronous stimulation in the S/L group. This depended crucially on the asynchronous signals being predictable (fixed temporal lag) and was eliminated on control conditions (vision-only, asynchronous-random), but it did not depend on the nature of the judgement (proprioceptive for RHI, visual appearance for EI). This suggests that the S/L group have differences in their internal model (i.e., less stringent assumptions about what is causal) that affects their experience of body ownership.

In other paradigms, perceivers have been shown to “recalibrate” lagging sensory signals to be consistent with an internal model of common cause (Fujisaki et al., 2004; Keetels & Vroomen, 2008; Parise et al., 2012). As such, what is unusual about the S/L group is the fact that they do so at larger temporal lags rather than the fact that they do this at all. Other research suggests that the RHI can be elicited at delayed visual feedback up to 300 ms but reduces thereafter (Shimada et al., 2009), and that this change in proprioceptive drift with delay does not follow the same pattern as perceptual judgements of delay (Shimada et al., 2014). It is currently unknown whether this difference is limited to causal inferences relating to body ownership (i.e., a domain-specific effect) or whether they would extend to other kinds of judgements such as audiovisual synchrony for non-bodily events (i.e., a domain-general effect) (for related research, see Costantini et al., 2016). Some researchers have speculated that mirror-sensory synaesthesia may be a result of domain-general differences in task control (Heyes & Catmur, 2015). It is also possible that the S/L group will be more likely to infer common cause over wider spatial as well as temporal windows. The RHI dissipates as the real and dummy hands are positioned further apart in space (Preston, 2013), but it is conceivable that this is not the case in the S/L group (consistent with the idea that they can feel touch/pain seen on other bodies at a distance).

The current study also compared a small group of participants who report vicarious pain that is non-localised and described more in terms of affective pain descriptors (termed A/G responders). The A/G group differed from the S/L in the asynchronous-predictable condition (both RHI and EI) and, overall, they tend to resemble the control group (Supplementary Material shows a combined analysis of data from this study and Botan et al., 2018b). A full consideration of this difference is beyond the scope of the present research, but one possibility is that the S/L group differs in processing of exteroceptive signals (whether limited to the body or not), whereas the A/G group reflects differences in the interoceptive domain. On other measures, the A/G and S/L groups appear similar such as reporting greater emotional contagion and more depersonalisation-like experiences (Botan et al., 2018a; Bowling et al., 2019).

This study highlights the importance of not only studying the modal pattern of behaviour but also considering variations in this. Although we have studied an atypical group, they are not exceptionally rare (12.3% in a sample of over 1,000; Botan et al., 2018b), and they were recruited from within standard psychology undergraduate cohorts. It is likely that all previous studies using the RHI and EI paradigms will have recruited and included some S/L responders unless, that is, they were excluded for producing unexpected results (i.e., no difference between synchrony and asynchrony). Whereas previous studies would have regarded data from these participants as noise, here we show that these differences within the “normal” population are coherent (i.e., resemble each other but differ from the norm) and meaningful (i.e., reflect some non-trivial difference in the underlying cognitive mechanisms).

The results of the present research also have important implications for our understanding of the mechanisms behind the RHI and EI. First of all, it is not the case that these illusions are driven by synchrony. In the case of the RHI, the mere presence of a dummy hand can drive proprioceptive drift. If anything, it is the presence of strongly contradictory signals (e.g., our asynchronous-random condition) that disrupt the illusion as suggested by Rohde et al. (2011). Second, we show a dissociation between proprioceptive drift and subjective illusion (questionnaires) on two different measures. Drift and subjective ratings may depend on different mechanisms in the brain (for a review, see Serino et al., 2013). The S/L group showed greater proprioceptive drift on the asynchronous condition that was not reflected in subjective ratings for that condition. Moreover, all groups showed greater proprioceptive drift on the vision-only condition that was not reflected in reports of illusory experiences for that condition. Interpretation of the first finding requires some caution because the previous study by Botan et al. (2018b) did find higher subjective ratings for the S/L group, and this group showed some evidence of higher subjective ratings in the EI.

Another novel contribution to this literature is the introduction of a measure of proprioceptive imprecision (variability in knowing the position of one’s limb prior to any experimental manipulations). The initial hypothesis was that people with greater proprioceptive imprecision would be more susceptible to the RHI (because they would weight vision more strongly). The evidence here was mixed. There was a positive correlation between proprioceptive imprecision and drift amplitude in the two most ambiguous conditions (asynchronous-predictable and vision-only), and this was led by the S/L group. Thus, this group is more sensitive to their own proprioceptive abilities in addition to other differences in their causal inferences (noting again that proprioception is irrelevant to the EI task where they also show a group difference).

In summary, the present study further explored susceptibility of vicarious pain responders to bodily ownership illusions by employing both RHI and EI paradigms. S/L responders display atypical susceptibility to bodily ownership illusions by treating asynchronous but predictable visuo-tactile signals as reflecting a common cause (i.e., stimulation to a single body).

## Supplemental Material

sj-docx-1-qjp-10.1177_17470218211024822 – Supplemental material for Vicarious pain is an outcome of atypical body ownership: Evidence from the rubber hand illusion and enfacement illusion Click here for additional data file. Supplemental material, sj-docx-1-qjp-10.1177_17470218211024822 for Vicarious pain is an outcome of atypical body ownership: Evidence from the rubber hand illusion and enfacement illusion by Vanessa Botan, Abigail Salisbury, Hugo D Critchley and Jamie Ward in Quarterly Journal of Experimental Psychology

sj-xlsx-2-qjp-10.1177_17470218211024822 – Research Data for Vicarious pain is an outcome of atypical body ownership: Evidence from the rubber hand illusion and enfacement illusion Click here for additional data file. Research Data, sj-xlsx-2-qjp-10.1177_17470218211024822 for Vicarious pain is an outcome of atypical body ownership: Evidence from the rubber hand illusion and enfacement illusion by Vanessa Botan, Abigail Salisbury, Hugo D Critchley and Jamie Ward in Quarterly Journal of Experimental Psychology This article is distributed under the terms of the Creative Commons Attribution 4.0 License ( https://creativecommons.org/licenses/by/4.0/) which permits any use, reproduction and distribution of the work without further permission provided the original work is attributed as specified on the SAGE and Open Access pages ( https://us.sagepub.com/en-us/nam/open-access-at-sage).

sj-xlsx-3-qjp-10.1177_17470218211024822 – Research Data for Vicarious pain is an outcome of atypical body ownership: Evidence from the rubber hand illusion and enfacement illusion Click here for additional data file. Research Data, sj-xlsx-3-qjp-10.1177_17470218211024822 for Vicarious pain is an outcome of atypical body ownership: Evidence from the rubber hand illusion and enfacement illusion by Vanessa Botan, Abigail Salisbury, Hugo D Critchley and Jamie Ward in Quarterly Journal of Experimental Psychology This article is distributed under the terms of the Creative Commons Attribution 4.0 License ( https://creativecommons.org/licenses/by/4.0/) which permits any use, reproduction and distribution of the work without further permission provided the original work is attributed as specified on the SAGE and Open Access pages ( https://us.sagepub.com/en-us/nam/open-access-at-sage).
