# Supplementary material for: Vicarious pain is an outcome of atypical body ownership: Evidence from the rubber hand illusion and enfacement illusion
Source: Q J Exp Psychol (Hove). 2021 Jun 29;74(11):1888–99. doi: 10.1177/17470218211024822 (PMC8450990; doi:10.1177/17470218211024822)
Supplement: sj-docx-1-qjp-10.1177_17470218211024822 – Supplemental material for Vicarious pain is an outcome of atypical body ownership: Evidence from the rubber hand illusion and enfacement illusion [file sj-docx-1-qjp-10.1177_17470218211024822.docx]

Supplementary Materials for:

**Vicarious Pain is an Outcome of Atypical Body Ownership:**

**Evidence from the Rubber Hand Illusion and Enfacement Illusion**

*V. Botan, Abigail Salisbury, H.D. Critchley, & J. Ward*

S1.1. RHI Questionnaire. Items and Subscales.

| **Subscale** | **Items** |
| --- | --- |
| **Ownership** | *It seemed like…* |
|  | 1. …I was looking directly at my own hand, rather than at a rubber hand. |
|  | 2. …the rubber hand began to resemble my real hand. |
|  | 3. …the rubber hand belonged to me. |
|  | 4. …the rubber hand was my hand. |
|  | 5. …the rubber hand was part of my body. |
| **Location** | 6. …my hand was in the location where the rubber hand was. |
|  | 7. …the rubber hand was in the location where my hand was. |
|  | 8. …the sensation I felt was caused by the paintbrush touching (or laser pointer playing on) the rubber hand. |
| **Agency** | 9. …I could have moved the rubber hand if I had wanted. |
|  | 10. …I was in control of the rubber hand. |

S1.2. EI Questionnaire. Items and Subscales.

| **Subscale** | **Items** |
| --- | --- |
| **Ownership** | 1. I felt the touch delivered in the other’s face. |
|  | 2. The touch I felt was caused by the cotton bud touching the other’s face. |
|  | 3. The other’s face was my face. |
|  | 4. The other’s face was part of my body. |
|  | 5. The other’s face belonged to me. |
| **Appearance** | 6. I was looking at my own reflection in a mirror rather than at the other’s face. |
|  | 7. The other’s face began to resemble my own face in terms of shape. |
|  | 8. The other’s face began to resemble my own face in terms of skin tone.  9. The other’s face began to resemble my own face in terms of facial features. |
| **Agency** | 10. The other’s face would have moved if I had moved. |
|  | 11. I was in control of the other’s face.  12. My own face was out of my control. |
| **Disownership** | 13. I couldn’t really remember how my face was. |
|  | 14. The experience of my own face was less vivid than normal. |

**Supplementary Results**

**Rubber Hand Illusion (RHI)**

**Proprioceptive Drift**

Table S2.1. Proprioceptive drift means ± standard deviations in mm for each condition and in each group.

|  | **Synchronous** | **Asynchronous** | **Visual Only** | **Asynchronous Random** |
| --- | --- | --- | --- | --- |
| **Controls** | 28.83 ± 33.04 | -1. 42 ± 16.21 | 17.65 ± 27.45 | 2.87±12.66 |
| **S/L** | 21.02±26.99 | 17.63±19.37 | 18.14±34.89 | -3.75±13.77 |
| **A/G** | 10.76±20.02 | 0.00±19.27 | 18.94±15.69 | 4.09±18.37 |

Table S2.2. Pearson correlations between proprioceptive imprecision and proprioceptive drift broken down by groups and conditions.

|  | Whole sample | Controls | S/L | A/G |
| --- | --- | --- | --- | --- |
| Synchronous | .087 | .094 | .064 | -.102 |
| Asynchronous | **.301*** | .053 | **.716**** | **-.666*** |
| Vision only | **.444**** | -.052 | **.731**** | .270 |
| Asynchronous-Random | .097 | .162 | -.158 | .395 |

Figure S2.1 Pooled results from the present and study and the previous study of Botan et al. (2018) that used identical methods for these conditions. The final sample size was: controls, N= 84; S/L, N= 41; A/G, N= 31.


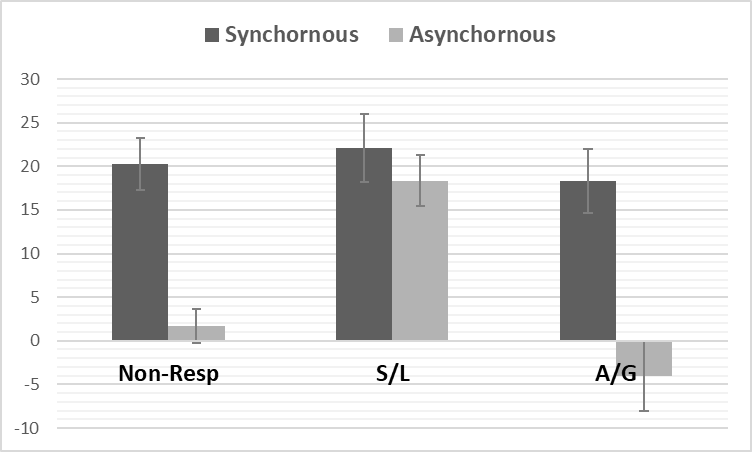


**Questionnaire results**

Table S2.3. Kruskal-Wallis H test results indicating group differences in RHI subjective ratings for each condition and each subscale.

|  | **Ownership** | **Location** | **Agency** |
| --- | --- | --- | --- |
| **Synchronous** | H= 0.770  p= 0.681 | H= 0.050  p= 0.975 | H= 0.802  p= 0.670 |
| **Asynchronous** | H= 2.238  p= 0.372 | H= 4.125  p= 0.127 | H= 3.657  p= 0.161 |
| **Vision-only** | H= 0.287  p= 0.866 | H= 0.752  p= 0.687 | H= 0.166  p= 0.920 |
| **Asynchronous- Random** | H= 0.905  p= 0.920 | H= 1.724  p= 0.422 | H= 4.330  p= 0.115 |

1. Ownership

Non-parametric Friedman test indicated that there were significant differences between conditions, χ^2^ = 56.705, p<0.001.

Post-hoc Wilcoxon tests indicate that feelings of ownership were greater in the synchronous condition than in all other conditions: asynchronous, Z=-6.412, p<0.001; visual, Z= -5.892, p<0.001; asynchronous-random, Z=-5.739, p<0.001. Feelings of ownership in the visual condition were greater than in the asynchronous condition, Z=-2.199, p=0.028 but not than in the asynchronous random condition, Z=-1.125, =p=0.261. Ratings in the asynchronous random and asynchronous were comparable: Z= -0.942, p=-0.346.

1. Location

Non-parametric Friedman test indicated that there were significant differences between conditions, χ^2^ = 50.466, p<0.001.

Post-hoc Wilcoxon tests indicate that perceived location was greater in the synchronous condition than in all other conditions: asynchronous, Z=-5.862, p<0.001; visual, Z= -5.781, p<0.001; asynchronous-random, Z=-5.387, p<0.001. Perceived location in the visual condition did not differ from the asynchronous condition, Z=-0.615, p=0.539 nor asynchronous-random condition, Z=-775, =p=0.439. Location ratings in the asynchronous random and asynchronous were comparable: Z= -0.364, p=0.716.

1. Agency

Non-parametric Friedman test indicated that there were significant differences between conditions, χ^2^ = 88.101, p<0.001.

Post-hoc Wilcoxon tests indicate that feelings of agency were greater in the synchronous condition than in all other conditions: asynchronous, Z=-5.311, p<0.001; visual, Z= -6.587, p<0.001; asynchronous-random, Z=-4.700, p<0.001. Feelings of agency in the visual condition were greater than in the asynchronous condition, Z=-4.197, p<0.001 and asynchronous-random condition, Z=-4.858, p<0.001. Ratings in the asynchronous random and asynchronous were comparable: Z= -0.779, p=0.436.

**Enfacement Illusion**

Table S2.4. Means ± standard deviations expressed in percentages of the difference between post-induction PSE and baseline PSE for each condition and in each group.

|  | **Synchronous** | **Asynchronous** |
| --- | --- | --- |
| **Controls** | 5.93 ± 4.76 | 2.72 ± 4.92 |
| **S/L** | 5.07 ± 6.84 | 5.11 ± 3.45 |
| **A/G** | 4.50 ± 4.67 | -0.50 ± 2.13 |

Table S2.5. Kruskal-Wallis H test results indicating group differences in EI subjective ratings for each condition and each subscale.

|  | **Ownership** | **Appearance** | **Agency** | **Disownership** |
| --- | --- | --- | --- | --- |
| **Synchronous** | H= 1.279  p= 0.528 | H= 1.953  p= 0.377 | H= 5.613  p= 0.060 | H= 3.956  p= 0.174 |
| **Asynchronous** | H= 3.495  p= 0.174 | H= 3.336  p= 0.189 | H= 1.974  p= 0.373 | **H= 7.040**  **p= 0.030** |
